# Supplementary material for: Single Cell High Dimensional Analysis of Human Peripheral Blood Mononuclear Cells Reveals Unique Intermediate Monocyte Subsets Associated with Sex Differences in Coronary Artery Disease
Source: Int J Mol Sci. 2024 Mar 1;25(5):2894. doi: 10.3390/ijms25052894 (PMC10932111; doi:10.3390/ijms25052894)

Supplemental Figure S1

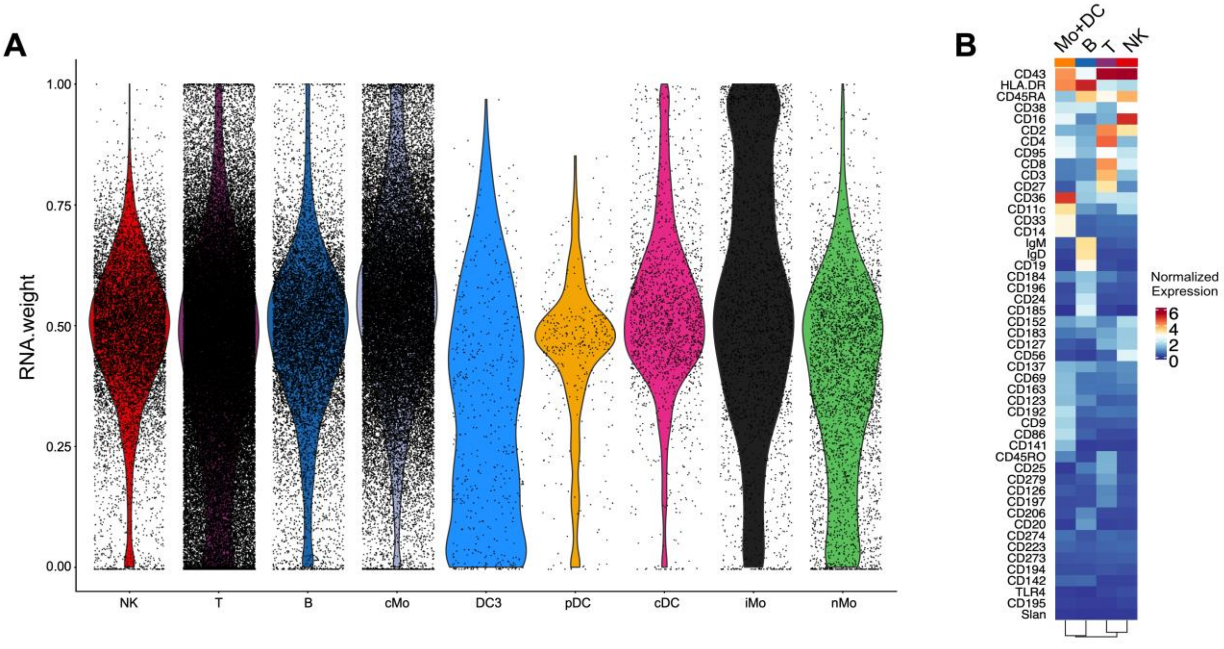

Supplemental Figure S2

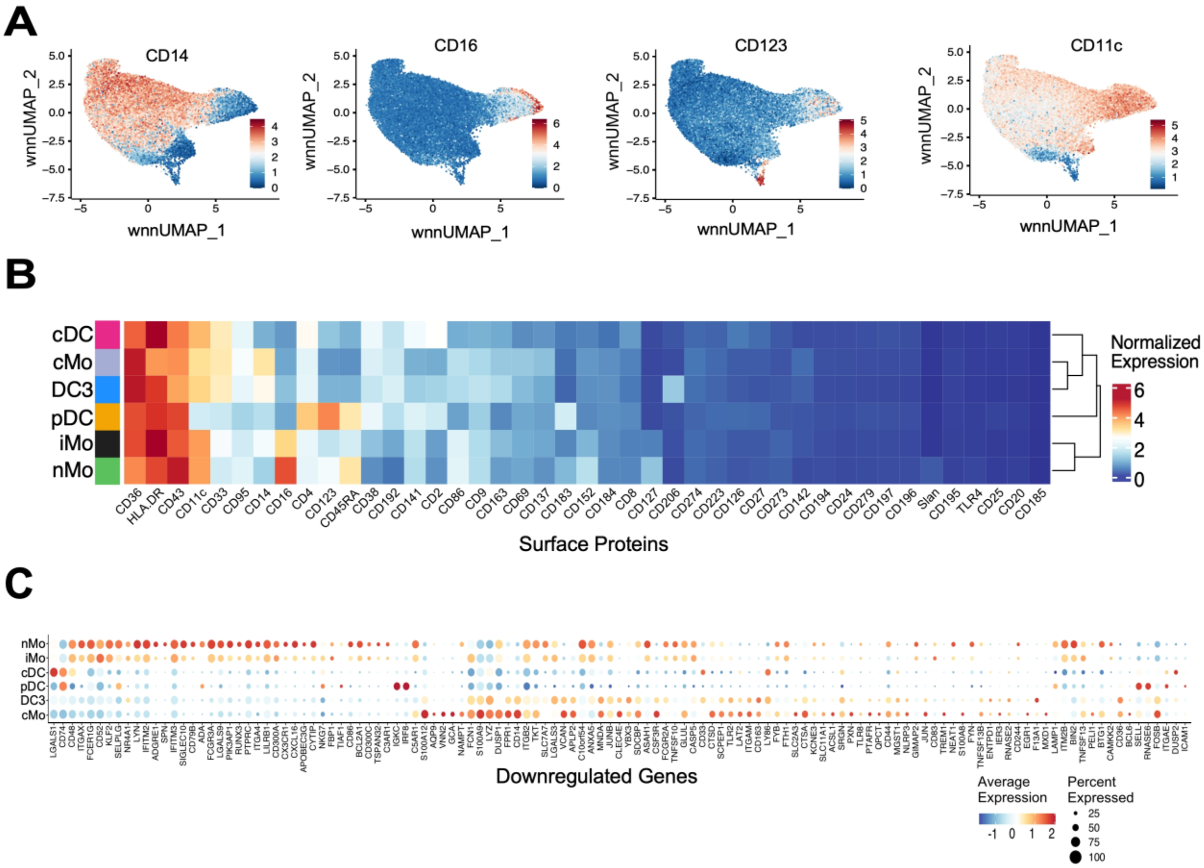

Supplemental Figure S3

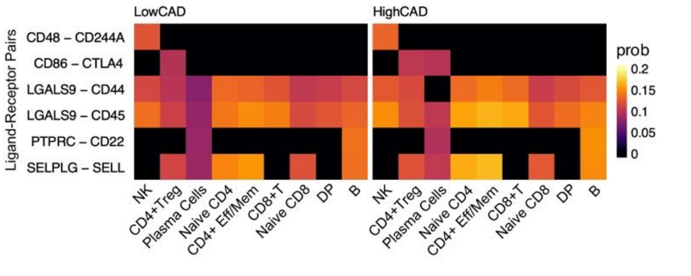

Supplemental Figure S4

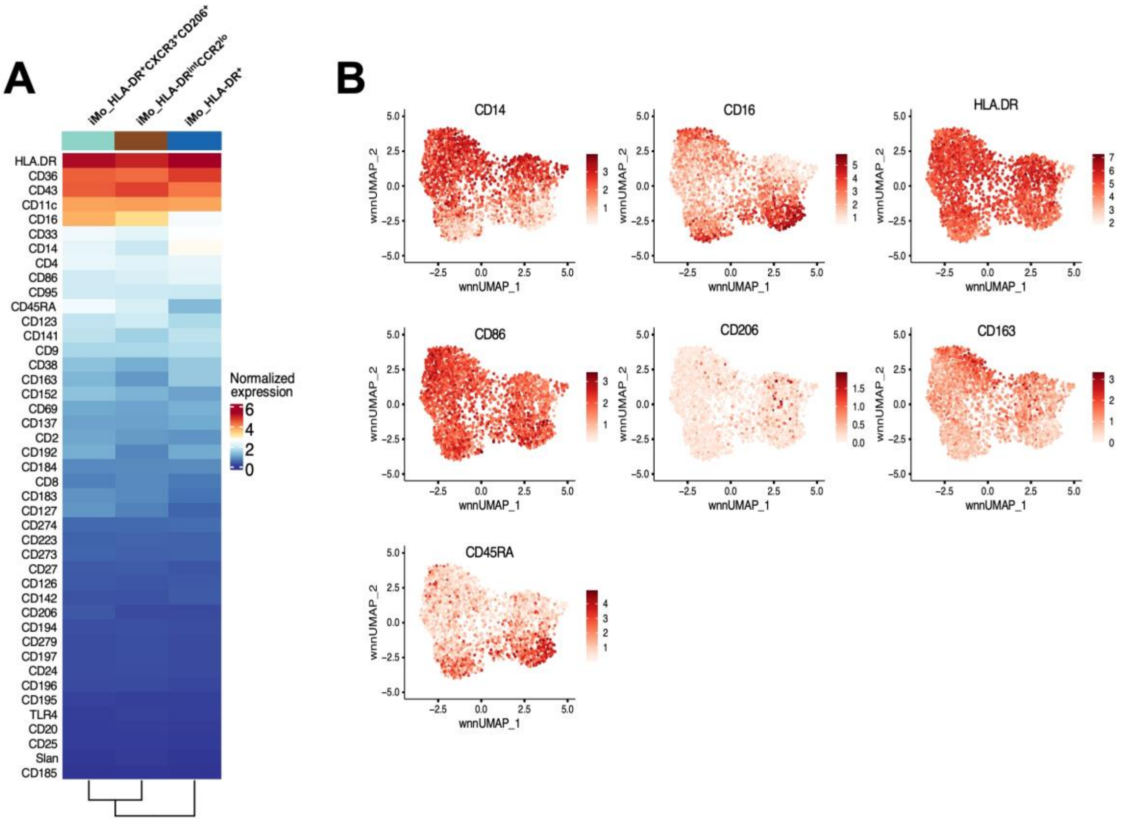

Supplemental Figure S5

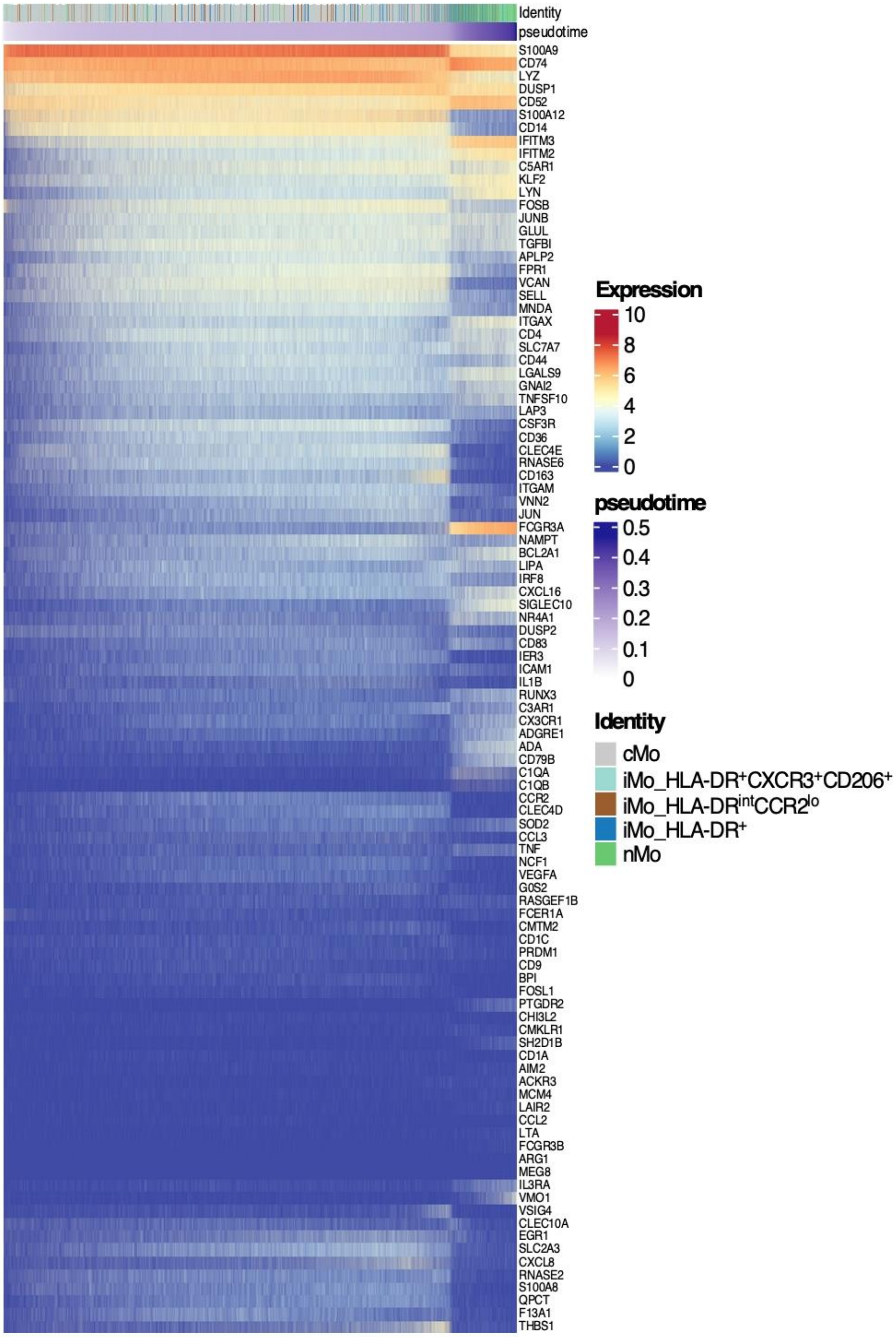

Supplemental Figure S6

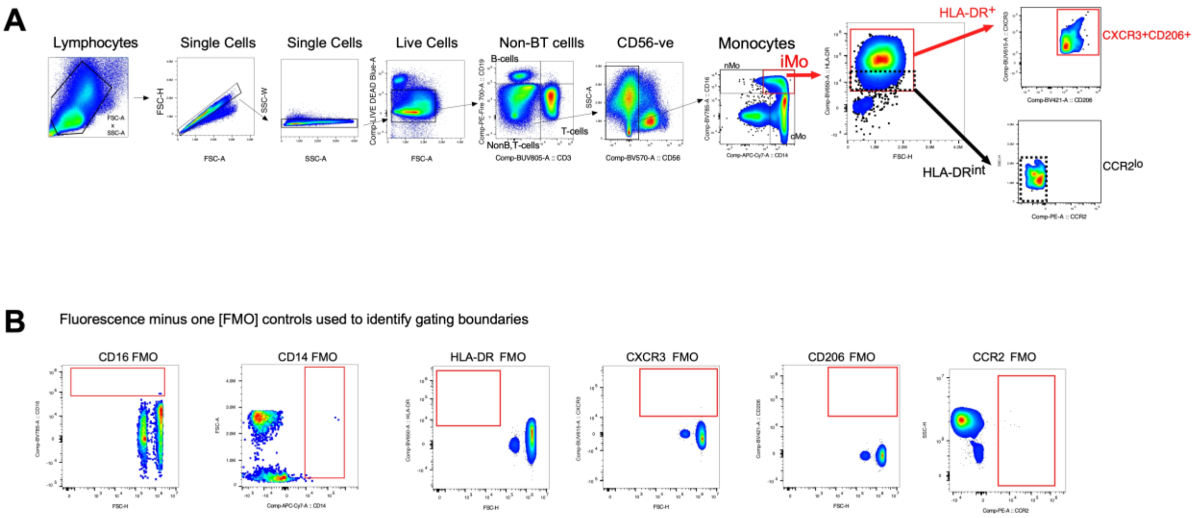

Supplemental Figure S7

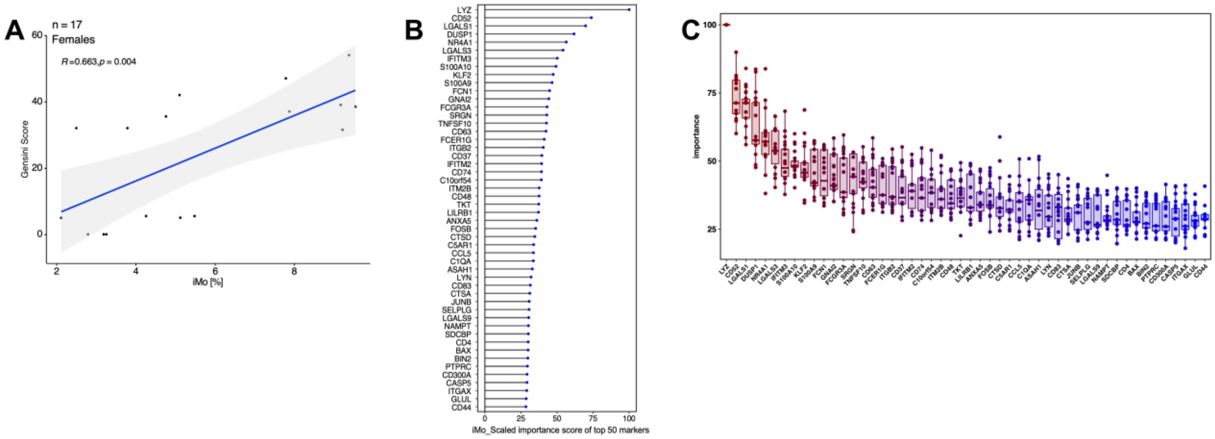

Supplement: Supplementary file 1 [file ijms-25-02894-s001.zip › FINAL_Supplemental Figures.pdf]
