# Supplementary material for: Single Cell High Dimensional Analysis of Human Peripheral Blood Mononuclear Cells Reveals Unique Intermediate Monocyte Subsets Associated with Sex Differences in Coronary Artery Disease
Source: Int J Mol Sci. 2024 Mar 1;25(5):2894. doi: 10.3390/ijms25052894 (PMC10932111; doi:10.3390/ijms25052894)
Supplement: Supplementary file 1 [file ijms-25-02894-s001.zip › FINAL_Supplementary Table S1.pdf]

Antibody Name	Company	Catalogue No.	Clone
1. L/D Blue	Invitrogen	L34962	
2. huCD3 BUV805	BD Biosciences	612895	UCHT1
3. huuCD14 APC-cy7	Biolegend	325620	HCD14
4. hCD16 BV785	Biolegend	302046	3G8
5. huCD19 PE/Fire 700	Biolegend	302276	HIB19
6. huCD56 BV570	Biolegend	362540	5.1H11
7. huHLA-DR BV650	Biolegend	307650	L243
8. huCD86 AF647	Biolegend	305416	IT2.2
9. huCD45RA BV510	Biolegend	304142	HI100
10. huCD206 BV421	Biolegend	321126	15-2
11. huCD183 (CXCR3) BUV615	BD Biosciences	751126	LS177-1C6
12. huCD192 (CCR2)	Biolegend	357206	K036C2
